# The Carbon Balance of a Rewetted Minerogenic Peatland Does Not Immediately Resemble That of Natural Mires in Boreal Sweden

**DOI:** 10.1111/gcb.70169

**Published:** 2025-04-08

**Authors:** Cheuk Hei Marcus Tong, Matthias Peichl, Koffi Dodji Noumonvi, Mats B. Nilsson, Hjalmar Laudon, Järvi Järveoja

**Affiliations:** ^1^ Department of Forest Ecology and Management Swedish University of Agricultural Sciences Umeå Sweden

**Keywords:** carbon cycle, climate change mitigation, environmental response functions, greenhouse gas emissions, net ecosystem carbon balance, peatland restoration

## Abstract

Rewetting is considered a strategy for mitigating carbon dioxide (CO_2_) emissions from drained peatlands, with associated climate benefits often derived by applying emission factors (EFs). However, data from rewetted sites are lacking, particularly for boreal peatland forests established on drained nutrient‐poor fens. Instead, their EFs have been developed primarily based on data from natural mires, implying similar carbon (C) cycles. In this study, we integrated eddy covariance measurements of ecosystem CO_2_ and methane (CH_4_) exchanges with dissolved C export estimates to compare the net ecosystem C balance (NECB) of a recently rewetted minerogenic peatland and two nearby undisturbed fen‐type mires in northern Sweden. We found that the rewetted peatland was an annual C source with a mean NECB of +77 ± 34 g C m^−2^ year^−1^ (±SD) over the initial 3 years following rewetting. In comparison, the mires were nearly C neutral or a C sink with their 3‐year mean NECB ranging between +11 and −34 g C m^−2^ year^−1^. The net CO_2_ emission of the rewetted peatland declined to about half by the third year coinciding with an increase in gross primary production. Annual CH_4_ emissions from the rewetted peatland steadily increased but remained at 32% and 49% in the first and third year, respectively, relative to the mires. We further noted differences in key environmental response functions of CO_2_ and CH_4_ fluxes between the rewetted and natural peatlands. Relative to the mires, the dissolved C loss was significantly greater in the rewetted peatland during the first year, but similar in subsequent years. Thus, our study demonstrates that the C balance of a recently rewetted minerogenic peatland may not immediately resemble that of natural mires. This further highlights the need for separate and dynamic EFs to improve estimates of the short‐term climate benefit of rewetting measures.

## Graphical abstract

This study assessed the carbon balance of a rewetted minerogenic peatland and two natural mires in northern Sweden using eddy covariance and discharge measurements. During 3 years after ditch‐filling, the rewetted site emitted +77 ± 34 g C m^−2^ year^−1^, while mires ranged between +11 and −34 g C m^−2^ year^−1^. CO_2_ emissions from the rewetted site decreased by half over the 3 years, while CH_4_ emissions increased toward 49% of mire levels. Dissolved carbon export differed only in the first year. This highlights the different carbon dynamics of recently rewetted and natural peatlands, calling for dynamic emission factors for rewetted peatlands.

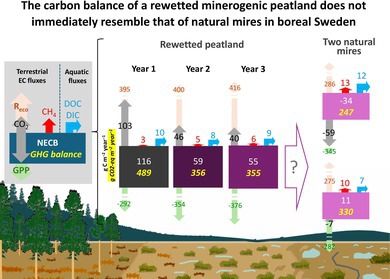

## 1 Introduction

Northern peatlands provide an important global carbon (C) pool, storing about twice as much C as all global forests combined (Leifeld and Menichetti 2018). During the past century, however, large areas of these peatlands have been drained for forestry, agriculture, and peat extraction (Vasander et al. 2003). Specifically, across the northern hemisphere, nearly 500,000 km^2^ (or ~15%) of mires have been drained for human use (Joosten and Clarke 2002) with some of the largest losses occurring in peatland‐rich countries such as Finland and Sweden where more than half of natural peatlands have been drained (Hånell 1990; Paavilainen and Päivänen 1995). The significant loss of C through enhanced oxidation following drainage has raised large concerns regarding the negative climate impact of these areas (Jones et al. 2017; Maljanen et al. 2010; Ojanen et al. 2013).

To mitigate C losses, the rewetting of drained peatlands (i.e., via ditch‐blocking or ditch‐filling; Landry and Rochefort 2012) has been proposed as an effective strategy to slow down the rate of peat decomposition and associated C emissions (Hiraishi et al. 2014; Joosten et al. 2016). Peatland restoration projects have been increasingly planned and implemented in Fennoscandia as a strategy for meeting EU environmental objectives (Gong et al. 2022; Noebel 2023). At the policy level, the climate benefit of rewetting measures is commonly estimated by the use of emission factors (EFs) for drained and rewetted peatlands, which were developed by the IPCC (Hiraishi et al. 2014) and later updated by Wilson et al. (2016). However, empirical evidence for C and greenhouse gas (GHG) fluxes from rewetted boreal peatland forests established on drained minerogenic mires has been limited. Instead, their EFs are primarily derived from data obtained from natural mires and rewetted bogs that were drained for peat extraction, implying similarity between the biogeochemistry of these systems (Hiraishi et al. 2014). This simplified assumption has been widely applied in governmental reports on emission accounting (e.g., UNFCCC 2024; Jordbruksverket 2018).

More recently, however, the use of these simplified EFs has been questioned. For instance, a recent study indicated that rewetted peatlands may remain functionally different from undisturbed mires for up to three decades (Kreyling et al. 2021). In addition, studies in temperate regions exploring the C balance following the rewetting of bogs drained for peat extraction (Nugent et al. 2018; Wilson et al. 2022) or forestry (Hambley et al. 2019) and of a fen drained for grassland use (Kalhori et al. 2024) suggested that net ecosystem CO_2_ losses may occur for at least one decade following rewetting. Furthermore, radiative forcing modeling revealed that the initial increase in methane (CH_4_) emissions following rewetting can delay anticipated climate benefits for several decades (Ojanen and Minkkinen 2020). Altogether, this indicates that the application of simplified EFs may considerably overestimate the initial climate benefit of peatland rewetting. Thus, comprehensive assessments of the C cycle in recently rewetted peatlands are needed, specifically for nutrient‐poor fens drained for forestry in the boreal region, for more accurately evaluating the effectiveness of rewetting as a strategy for mitigating climate change.

Differences in the net ecosystem CO_2_ exchange (NEE) of natural mires, rewetted peat extraction sites, and rewetted peatland forests might be caused by contrasting hydrology, peat decomposition rates, and/or plant biomass production. Both water balance and C cycle processes are commonly at steady‐state conditions in natural systems while undergoing a transformation phase following rewetting. For instance, Andersen et al. (2013) showed that peat microbial communities and associated decomposition rates differed between natural and rewetted peatlands. Furthermore, the recovery rates of the *Sphagnum* community within the first decade following rewetting were reported to be highly variable depending on nutrients and water table level (WTL) (Maanavilja et al. 2015; Komulainen et al. 1999; Tuittila et al. 2000b). While our current knowledge of rewetting effects on the C balance relies primarily on studies in restored bogs drained for peat extraction (Petrone et al. 2001; Järveoja et al. 2016; Nugent et al. 2018; Strack and Zuback 2013), differences can be expected in rewetted fens that were drained for forestry. Specifically, the latter have been subject to over a century of drainage, which may lead to significant changes in soil structure and hydrological properties (Turunen et al. 2024; Menberu et al. 2021), nutrient dynamics (Nieminen et al. 2021), and C cycling (Dubra et al. 2023). Furthermore, while the photosynthetic ground vegetation and upper well‐decomposed peat layer are removed during peat extraction, these are maintained in peatland forests, likely resulting in different plant C uptake and peat decomposition rates after rewetting. On the other hand, rewetting of drained forested peatlands often involves clearcutting of the trees, which may also disturb the plant community of the ground vegetation (Maanavilja et al. 2014) and possibly enhance heterotrophic respiration due to the decay of fresh organic matter in the form of harvest residuals and root biomass (Korkiakoski et al. 2019). Furthermore, water and nutrient inflow from the surrounding upland areas might modify the rewetting process and C cycle responses in minerogenic peatlands. Given these system‐specific transformative processes following rewetting, there is a need to better understand the initial dynamics in NEE, in particular for rewetted minerogenic peatlands.

One concern about rewetting is that CH_4_ emissions commonly increase in response to the elevated WTL (Bubier et al. 1995; Jordan et al. 2016). However, CH_4_ emissions from rewetted peatlands may remain different from those in undisturbed mires (Komulainen et al. 1998; Jordan et al. 2016). Several studies conducted in temperate nutrient‐rich regions reported that CH_4_ emissions from rewetted sites may even exceed those from natural peatlands (e.g., Hahn‐Schöfl et al. 2011; Vanselow‐Algan et al. 2015). Furthermore, there is considerable uncertainty about the recovery rates of CH_4_ emissions following rewetting, which may vary in dependence of the methanogen community development (Laine et al. 2019) and vegetation establishment, with the latter regulating substrate supply (Urbanová and Bárta 2020) and CH_4_ emission via transport through aerenchymatic plant tissue (Bārdule et al. 2023; Komulainen et al. 1998). Given that CH_4_ has a 27‐fold higher global warming potential (GWP) than CO_2_ over a 100‐year timeframe (Lee et al. 2023), even small differences in CH_4_ emission may therefore substantially modify the climate impact of rewetting measures.

To date, most studies on CO_2_ and CH_4_ fluxes in rewetted peatlands rely on bi‐weekly to monthly chamber measurements during the growing season (e.g., Koskinen et al. 2016; Purre et al. 2019; Strack et al. 2014), which require model extrapolation to estimate the annual balance. In contrast, the eddy covariance (EC) method directly quantifies the ecosystem‐scale GHG exchanges continuously at a high temporal resolution (i.e., half‐hourly) and all year round (Baldocchi 2003). This significantly reduces the need for extrapolation when estimating annual balances. Recent advances in fast‐response CH_4_ analyzers now enable the use of the EC method to quantify also CH_4_ fluxes alongside the CO_2_ fluxes (Saha et al. 2024), thus providing a more comprehensive and accurate assessment of the full C and GHG balances of these ecosystems. At present, however, EC flux estimates are still lacking primarily for rewetted minerogenic peatlands in the boreal region (Escobar et al. 2022).

Apart from the terrestrial C fluxes, the aquatic export of dissolved organic and inorganic C (DOC, DIC) may constitute another important component of the peatland C balance (Roulet et al. 2007; Nilsson et al. 2008). Factors such as soil fertility, hydrology, and vegetation cover have been identified as key determinants influencing the export of DOC and DIC (Leach et al. 2016; Moore et al. 2002). These factors, and hence the EFs derived for dissolved C, could vary considerably between rewetted and natural peatlands. However, previous studies provided comparisons solely between rewetted and drained conditions (e.g., Kaila et al. 2016; Koskinen et al. 2017; Strack et al. 2015), whereas comparisons between rewetted and natural peatlands are lacking. Furthermore, most studies focus on investigating only the concentrations of dissolved C (Kaila et al. 2016; Strack et al. 2015), without combining them with discharge measurements to estimate the aquatic C export. Thus, there is a need for empirical data on dissolved C export from rewetted peatlands for comparison with that of natural mires, and to improve our understanding of the total C balance of rewetted peatlands.

The concept of the Net Ecosystem Carbon Balance (NECB) integrates C fluxes from both terrestrial and aquatic environments to assess whether ecosystems act as a net sink or source of C (Chapin et al. 2006). While the NECB has been previously estimated for natural mires (Koehler et al. 2011; Nilsson et al. 2008; Roulet et al. 2007) and rewetted bogs in temperate regions drained for peat extraction (Nugent et al. 2018; Wilson et al. 2022), equivalent data for rewetted minerogenic peatlands in the boreal region remain scarce. However, since most of the drained and forested peatland areas in Fennoscandia were established originally on fens, a better understanding of how rewetting alters the NECB of these peatlands is of particular value.

In this study, we combined EC measurements of terrestrial CO_2_ and CH_4_ fluxes with dissolved C export estimates with the aim to compare the C dynamics of a recently rewetted minerogenic forested peatland and two nearby undisturbed mires in boreal Sweden. The specific objectives were to compare: (i) their annual NECB, (ii) the relative contributions of the individual NECB component fluxes, and (iii) the responses of the NECB component fluxes to key environmental variables.

## 2 Materials and Methods

### 2.1 Site Description

**FIGURE 1:**
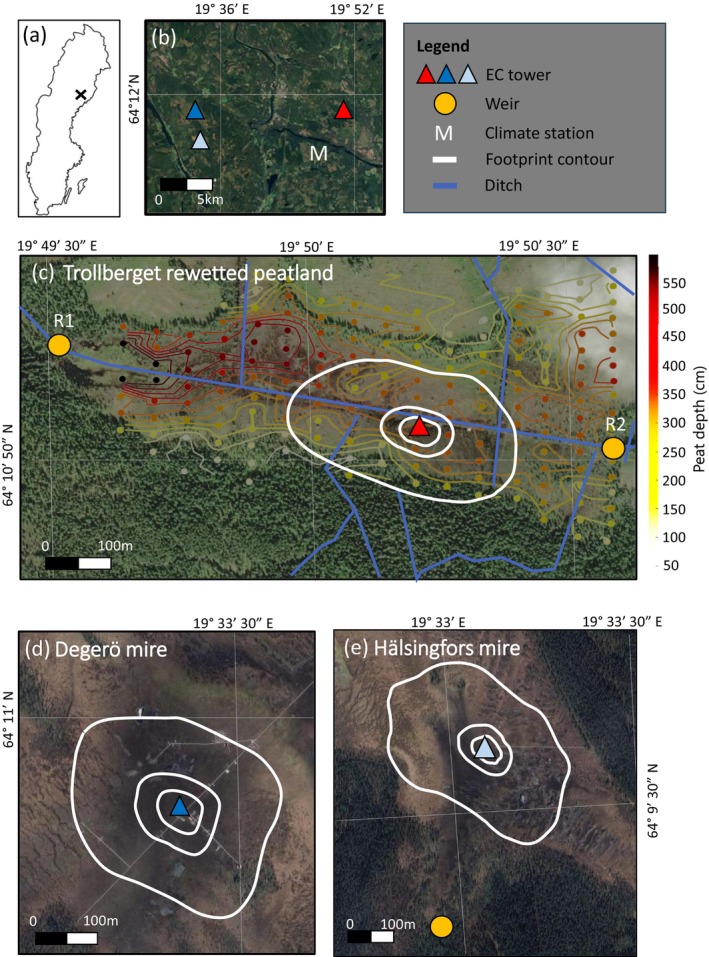
(a) Location of the study sites in Sweden, (b) locations of the Trollberget rewetted peatland (red triangle), the Degerö mire (dark blue triangle), and the Hälsingfors mire (light blue triangle), and (c‐e) experimental setup of the three sites. In (b), the letter M denotes the location of the long‐term climate station Vindeln‐Sunnansjönäs. In (c‐e), the triangles denote the location of the eddy covariance (EC) towers. The blue lines denote the ditch network. The white contours denote the 50%, 70%, and 90% footprint contours of the EC system based on the Kljun et al. (2015) model. In (c), the yellow circles denote the weirs for discharge measurements and water sample collections. The background pattern of dots and contour lines represents peat depth sampling points and modeled gradients, with colors indicating depth. Note that the measurement and sampling weir at Degerö is located 1.0 km from the EC system and thus not illustrated in the figure. Map lines in panel (a) delineate the study area and do not necessarily depict accepted national boundaries.

This study was conducted at the Trollberget Experimental Area (TEA; Laudon et al. 2021), (64°10′51.60″N, 19°50′14.08″ E, 227 m a.s.l.), situated in the Västerbotten county, northern Sweden, approximately 45 km northwest of Umeå (Figure 1). The climate of the region is characterized by a 30‐year mean (1991–2020) air temperature of +3.0°C and annual precipitation of 635 mm based on data from the nearest (i.e., 6.7 km) national reference climate station Vindeln‐Sunnansjönäs (64°08′13.56″N, 19°46′19.16″ E) operated by the Swedish Meteorological and Hydrological Institute (SMHI; https://www.smhi.se). The length of the growing season (with the start and end dates defined as the first day out of five consecutive days with daily mean air temperature > 5°C and < 5°C, respectively) was 155 days averaged over the study years.

The study site was originally a sparsely treed minerogenic oligotrophic mire with characteristics of a flark fen consisting of a series of alternating flark (i.e., hollow) and string (i.e., peat ridge) patterns in its central parts. The mire was likely drained in the few years following 1910 (when the legal application for carrying out the ditching was formally documented), i.e., about 110 years ago (Norstedt et al. 2021; pers. comm.). The ditch network was dug by hand and consisted of a ~5 m wide central main ditch running east–west across the site, connected to several secondary ~1 m wide ditches running in the north–south direction. The site is drained by two outlets (R1 and R2; Figure 1c), which receive runoff from catchment areas of 47 and 60 ha, respectively (Laudon et al. 2021). Following drainage, the originally treed areas (located primarily on the northern and eastern parts of the site) developed into a low‐productive peatland forest (dominated by *Pinus sylvestris* ; basal area = 2.6 m^2^ ha^−1^) as described on historic maps from 1924 (Norstedt et al. 2021; pers. comm.). In November 2020, the area was rewetted using a 20‐ton crawling excavator following conventional authority‐defined methods (Laudon et al. 2023). More specifically, the trees on the site were harvested and the existing drainage ditches were filled with on‐site peat and the harvested tree logs. The branches and slash (5.5 t dry biomass ha^−1^) from tree harvesting were left on the site.

As no active restoration was undertaken beyond ditch‐blocking and ditch‐filling, the vegetation cover currently resembles primarily residual plant communities which were already present before rewetting. The vegetation composition within the EC flux footprint area at the time of the study consisted of both vascular plants as well as *Sphagnum* spp. mosses. Specifically, the dominant shrubs included species such as common heather ( *Calluna vulgaris* L. (Hull)), black crowberry ( *Empetrum nigrum* L.), bog rosemary ( *Andromeda polifolia* L.), and *Vaccinium* spp., while the main graminoid species was cottongrass ( *Eriophorum vaginatum* L.). The site also includes a few areas of bare peat and open water. Peat depth probing conducted at the site in 2019, i.e., before rewetting, indicated that the mean peat depth was 241 cm (ranging from 22 to > 599 cm; *n* = 190; Table 1 and Figure 1). The mean peat C:N ratio was 45.5 ± 2.1 varying between 36 and 59 (Casselgård 2020; Table 1). The mean growing season WTL at the rewetted peatland during the study period was 11.3 cm below the surface (Table 1).

**TABLE 1:** Soil (0–35 cm depth) properties at the Trollberget rewetted peatland^a^ and the Degerö^b^ and Hälsingfors^b^ natural mires.

	Trollberget rewetted peatland	Degerö natural mire	Hälsingfors natural mire
Carbon (C, %)	51.7 ± 0.4	47.9 ± 0.2	52.0 ± 0.3
Nitrogen (N, %)	1.4 ± 0.1	0.8 ± 0.2	1.3 ± 0.1
C:N ratio	45.5 ± 2.1	66.5 ± 4.4	45.0 ± 2.6
Peat depth mean (range) (cm)	241 (22 to > 599)	217 (10–653)^c^	n.a.
Bulk density (g cm^−3^)	0.099 ± 0.005	0.038 ± 0.003	0.092 ± 0.004
Mean water table level (cm)	−11.3 ± 3.7	−8.5 ± 2.5	−8.2 ± 5.0

*Note:* Mean water table (with negative values indicating a position below the surface) represents the mean over the growing seasons 2021–2023. Numbers represent means ±1 standard error, n.a. indicates no data available.

^a^ Casselgård (2020).

^b^ Noumonvi et al. (2023).

^c^ Peng et al. (2024).

The Degerö and Hälsingfors mires are located within the Kulbäcksliden Research Infrastructure (Noumonvi et al. 2023), situated about 14 km from the TEA. These mires are oligo‐minerotrophic fens dominated by lawn and carpet plant communities (Nilsson et al. 2008; Tong et al. 2024), with an abundance of *Sphagnum* spp. mosses and graminoids including cottongrass, rannoch‐rush ( *Scheuchzeria palustris* L.), and tufted bulrush ( *Trichophorum cespitosum* (L.) Hartm.). Thus, they are classified as low‐sedge type mires which is the dominant peatland site type in boreal Sweden (Nilsson et al. 2001). Including two instead of only one mire site provided the possibility to consider variations within the mire reference. Specifically, the Degerö mire had a higher C:N ratio (66.5 ± 4.4) and lower bulk density (0.038 ± 0.003 g cm^−3^) than the Hälsingfors mire, with the latter having more similar soil characteristics compared with the Trollberget rewetted peatland (Table 1). The mean growing season WTL near the flux tower was 8.5 and 8.2 cm below the surface at the Degerö and Hälsingfors mires, respectively (Table 1). It remains elusive how similar the Trollberget mire was before drainage to these reference mires, however, given their similar climatic and geological context, post‐glacial developmental age, trophic levels, and land use of the surrounding areas, it is likely that they featured relatively similar peatland properties.

### 2.2 Net Ecosystem CO_2_ and CH_4_ Exchanges

Continuous measurements of net ecosystem CO_2_ and CH_4_ exchanges were conducted with the EC method. At the rewetted site, a CPEC300 EC flux system was installed at 2.5 m height to a mast located next to the main ditch near the center of the restored area in January 2021 (Figure 1c). Fluctuations of wind and air temperature were measured by a CSAT3 sonic anemometer (Campbell Scientific Inc., Logan, UT, USA), whereas fluctuations of CO_2_ concentration were measured by an EC155 closed‐path gas analyzer (Campbell Scientific Inc., Logan, UT, USA). The air inlet had a vertical separation of −10 cm and a horizontal separation of 26 cm with the anemometer. The air was drawn to the analyzer sample cell through a polypropylene tubing (Synflex 1300, 2.2 mm inner diameter, 64.5 cm length) at 7.5 L min^−1^. Fluctuations of CH_4_ concentration were measured by a LI‐7700 open‐path analyzer (Li‐COR Biosciences, NE, USA). Data from the sonic and gas analyzers were recorded on a CR6 data logger (Campbell Scientific Inc., Logan, UT, USA). The entire system was powered all year round by a combination of solar panels and an EFOY Pro 800 methanol fuel cell (SFC Energy, Brunnthal, Germany).

The high‐frequency (10 Hz) EC data were processed using the open‐source flux calculation software EddyPRO (version 7.0.4, Li‐COR Biosciences, NE, USA). Specifically, to align the sonic anemometer along local wind streamlines, double‐coordinate rotation was performed using the three wind velocity components (Wilczak et al. 2001). Linear trends were removed by block averaging and linear detrending over 30‐min averaging periods (Gash and Culf 1996). The automatic time lag optimization method (Rebmann et al. 2012) was utilized to determine time lags between vertical wind speed and gas concentration. Data were filtered to eliminate periods with low signal strength of EC instruments, as well as non‐steady state or low turbulent conditions (Foken and Leclerc 2004). A change‐point detection method (Barr et al. 2013) was used to determine friction‐velocity thresholds (0.05–0.09 m s^−1^) for moving time period windows to capture seasonal dynamics in turbulence conditions. These thresholds were applied to filter out data during low turbulent conditions. The processed data was averaged to half‐hourly means and data points that exceeded ±2 standard deviations from the 30‐day moving window mean for a given half‐hour were identified as statistical outliers and discarded. Following these initial quality control and filtering steps, 35% and 29% of all potential half‐hourly CO_2_ and CH_4_ values remained for the three study years, respectively (Figure S1).

Spectral correction for high (Moncrieff et al. 2004) and low (Ibrom et al. 2007) frequency losses was applied for all fluxes. Measured CH_4_ fluxes were corrected for spectroscopic effects along with compensation terms for air density fluctuations (Webb et al. 1980). This involves removal of the effects of temperature, pressure, and water vapor fluctuations using the method reported by McDermitt et al. (2011). Changes in CO_2_ storage below the analyzer intake height were accounted for by using the single‐point storage term calculation described in Aubinet et al. (2001).

At the Degerö mire, which is an ecosystem station (SE‐Deg; www.icos‐sweden.se/degero) within the pan‐European network of the Integrated Carbon Observation System (ICOS), fluxes were measured at 3.07 m above ground following the standard ICOS protocols. The instrumental setup consisted of a Metek uSonic‐3 Class A anemometer, a LI‐7200 gas analyzer for CO_2_ and H_2_O concentration, and LGR FGGA 911–0010 (Los Gatos Research, Mountain View, CA, USA) for CH_4_ concentration measurements. At the Hälsingfors mire, gas concentration was measured by a CO_2_‐CH_4_‐H_2_O Picarro G2311‐f analyzer, alongside a Metek uSonic‐3 Class A anemometer mounted at 2.75 m height at the site. At Degerö, a total of 55% and 58% of all potential half‐hourly CO_2_ and CH_4_ values, respectively, remained for the three sample years, whereas a total of 41% and 51% of all potential half‐hourly CO_2_ and CH_4_ values were retained at the Hälsingfors mire, respectively (Figure S1). More details regarding instrumentation at the Degerö and Hälsingfors mires were described in Noumonvi et al. (2023).

At each site, the final quality‐controlled half‐hourly fluxes of CO_2_ and CH_4_ were then gapfilled using machine learning models based on environmental input variables. Specifically, XGBoost was used to gap‐fill NEE according to Kämäräinen et al. (2023) and Vekuri et al. (2023), while random forests were used for gap‐filling the CH_4_ flux according to Irvin et al. (2021). The coefficient of determination (*R* ^2^) of predicted versus gap‐filled fluxes for holdout sets during the 10‐fold cross validations ranged between 0.89 and 0.94 for NEE, and between 0.76 and 0.94 for CH_4_ flux (Figure S3). Environmental variables used as predictors for gap‐filling the fluxes included air temperature, soil temperature (*T*_s_) at 10 cm depth (*T*_s10_), WTL, air pressure, photosynthetically active radiation (PAR), relative humidity, and vapor pressure deficit. In addition, indicators of the time of the year such as yearly sine, yearly cosine, and time delta (Irvin et al. 2021) were also used as predictors both for NEE and CH_4_ flux. The gapfilled NEE was further separated into ecosystem respiration (*R*_eco_) and gross primary productivity (GPP) based on the nighttime‐based partitioning method (Reichstein et al. 2005). The EC flux footprint was evaluated with the Flux Footprint Prediction model (Kljun et al. 2015).

### 2.3 Dissolved C Export via Stream Discharge

To estimate dissolved C export at the study sites, we installed V‐notch weirs equipped with automated water level sensors (Solinst Levelogger 5; Solinst Canada Ltd., Georgetown, ON, Canada) at the catchment outlets and conducted manual sampling of discharge rates and dissolved C concentrations. Since the rewetted site comprises two catchments, two V‐notch weirs were established at each end of the central main ditch—one to the west and one to the east of the flux footprint area (Figure 1c). Manual sampling campaigns were conducted 80, 72, and 73 times over the three study years at the rewetted site, the Degerö mire, and the Hälsingfors mire, respectively. Of these, 42%, 33%, and 33% were carried out during high‐flow conditions associated with snowmelt floods in April and May. Manual discharge measurements at the weirs were made with the volumetric gauging method, which were subsequently regressed against the hourly water level data to yield continuous estimates of the discharge rate.

During each sampling campaign, water samples were collected to calculate the concentration of dissolved C. The concentration of DOC was assessed through the utilization of a Shimadzu TOC‐CPCH analyzer (Ågren et al. 2007; Buffam et al. 2007). DIC was quantified using a headspace method following Wallin et al. (2013). Previous investigations conducted in boreal Swedish surface waters have suggested that the concentrations of particulate organic carbon (POC) are insignificant compared with the dissolved fraction (Laudon et al. 2011; Leach et al. 2016). However, disturbance during ditch‐filling might have resulted in increased POC levels in runoff immediately following rewetting. In this study, however, data on POC were not available to elucidate its response to rewetting and subsequent implication for the aquatic C export. Instead, the DOC concentration was assumed to be representative for the concentration of total organic C. After linear interpolation of the DOC and DIC concentrations to hourly intervals, we multiplied these with the discharge flow rate to determine DOC and DIC export.

### 2.4 Net Ecosystem Carbon Balance and Total GHG Emissions

We estimated the NECB based on the concept by Chapin et al. (2006), which describes the ecosystem C sink‐source strength after incorporating all vertical and lateral fluxes of organic and inorganic C. In our study, the NECB was determined from the sum of NEE, CH_4_ emissions, and the aquatic C export. We acknowledge that additional C input fluxes via precipitation and lateral C inflow from the surrounding upland areas, as well as C losses via POC export may further modify the NECB, however, we were not able to account for these in this study. The sign convention in this study is such that negative and positive fluxes represent C input (i.e., uptake) into and C output (i.e., emission or lateral export) from the ecosystem, respectively. It is important to note that due to that, our NECB estimates are negative when the ecosystem acts as a C sink (which is common in studies of peatland ecosystems, see, e.g., Nilsson et al. 2008), Roulet et al. (2007), but contrary to the original sign convention of NECB as defined by Chapin et al. (2006). To estimate the total annual GHG balances, the annual CH_4_ fluxes were transformed into CO_2_‐equivalents (CO_2_‐eq) by applying a global warming potential (GWP) of 27 over a 100‐year timeframe (Lee et al. 2023).

### 2.5 Environmental Variables

At the rewetted site, WTL and *T*_s_ (at 2, 10, 15, 30, and 50 cm depth) were continuously recorded by CS451 pressure transducers (Campbell Scientific Inc., Logan, UT, USA) and TO3R sensors (TOJO Skogsteknik Soil, Bygdeå, Sweden), respectively, at four separated locations around the tower (~15 m away) with each two pits on the north and south side of the main ditch (Figure 1c). The same WTL and *T*_s_ sensors were installed at two separated locations around the tower at the Hälsingfors mire, while Fischer Pt 100 *T*_s_ sensors and CS450 WTL sensors were installed at six replicated plots at the Degerö mire.

Ambient air temperature at 2 m above the surface was continuously measured with Rotronic MP102H‐331,000 at Degerö and HC2S3 probe at the other two sites. PAR was continuously recorded with a Li‐190 quantum sensor (Li‐Cor Inc., Lincoln, NE, USA) at all sites. Net radiation including its separate in‐ and outgoing short‐ and longwave components was measured with a CNR4 net radiometer at Degerö and NR01 net radiometer at the other two sites (Hukseflux Thermal Sensors B.V., the Netherlands). Data from these automated sensors were logged on CR1000 data loggers (Campbell Scientific Inc., Logan, UT, USA) at 1‐min intervals and stored as half‐hourly averages. Normalized difference vegetation index (NDVI) was determined for the EC footprint areas from remote sensing images by the Sentinel‐2 sensor (Drusch et al. 2012), providing 83, 90, and 96 images at the rewetted peatland, Degerö, and Hälsingfors sites, respectively, over the 3‐year measurement period.

## 3 Results

### 3.1 Environmental Conditions

**FIGURE 2:**
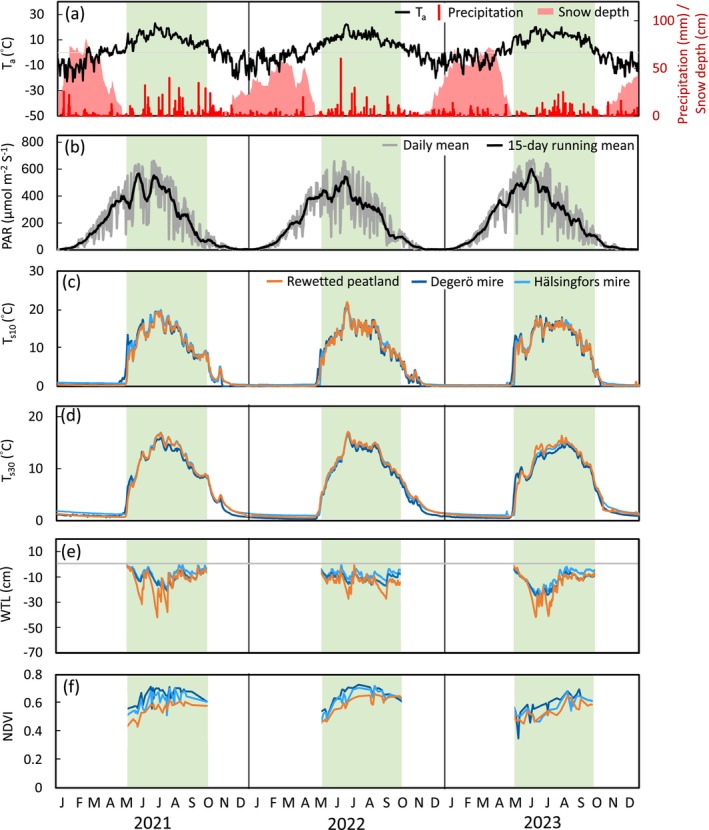
Daily means of environmental parameters at the Trollberget rewetted peatland and the two natural mires during the study period of 2021–2023. These include (a) air temperature (T_a_), precipitation and snow depth, (b) photosynthetically active radiation (PAR), (c) soil temperature at 10 cm (*T*_s10_), (d) soil temperature at 30 cm (*T*_s30_), (e) water table level (WTL), and (f) normalized difference vegetation index (NDVI). T_a_ data are presented as the average of the three sites, whereas precipitation, snow depth, and PAR data are retrieved from the Vindeln‐Sunnansjönäs climate station. WTL and NDVI data are presented during the growing season with available data. The green areas denote the growing season periods. Lines show the daily means of WTL averaged over 4, 2, and 6 sampling locations at the Trollberget rewetted peatland, Hälsingfors mire, and Degerö mire, respectively.

The average air temperature across the three study sites from 2021 to 2023 was +2.2, +3.3, and +2.6°C, being similar to the 30‐year long‐term (1991–2020) mean of +3.0°C (Figure 2a). The annual precipitation was 781, 573, and 577 mm for the years 2021, 2022, and 2023, being significantly above the 30‐year average (635 mm) in the first year, with the additional precipitation occurring primarily from June to October (Figure 2a). It is noteworthy that the early summer in 2023 was unusually dry, with May and June receiving only 41% of the precipitation compared with the average of the previous 2 years.

The 3‐year growing season means of *T*_s_ at 10 cm (*T*_s10_) and 30 cm (*T*_s30_) depth were within a limited range of 12.8°C–13.2°C and 11.4°C–11.9°C, respectively, across the three sites (Figure 2c,d). While growing season *T*_s_ remained similar across the three sites, *T*_s30_ during the peak growing season (June to August) and the early snow period (November to December) was about 1°C lower at the Degerö mire than at the other two sites. Despite the similar growing season mean WTL (Table 1), the seasonal range (−42 to 0 cm) at the rewetted peatland was larger compared with the Degerö (−27 to −2 cm) and Hälsingfors (−24 to +1 cm) mires. Specifically, whereas the WTL at the rewetted peatland was similar to the two mires during wet periods (WTL > −10 cm), significantly lower WTL was observed at the rewetted peatland during dry periods (Figure 2e). Among the three sites, the mean NDVI during the growing season was lowest at the rewetted peatland (0.57), compared with the Hälsingfors (0.61) and Degerö mires (0.65) (Figure 2f). The mean growing season NDVI at the rewetted peatland increased from 0.56 in 2021 to 0.60 in 2022. However, a decrease to 0.54 was noted in 2023, coinciding with the dry early summer that year. A similar reduction was also observed at the mires in 2023.

### 3.2 Temporal Variations of Ecosystem CO_2_ and CH_4_ Exchanges in Recently Rewetted and Natural Peatlands

**FIGURE 3:**
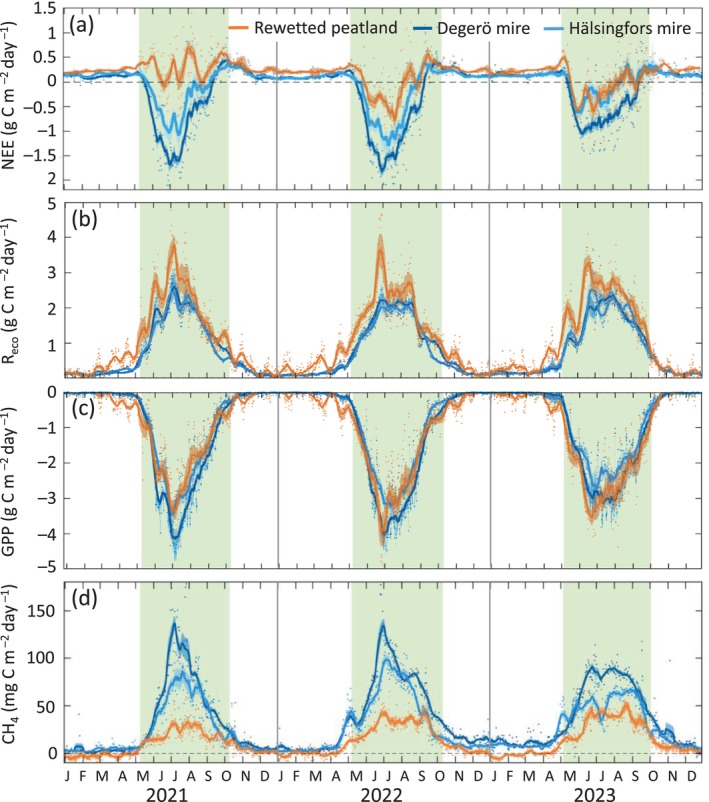
Daily sums of (a) net ecosystem CO_2_ exchange (NEE), (b) ecosystem respiration (*R*_eco_), (c) gross primary productivity (GPP), and (d) methane (CH_4_) flux for the Trollberget rewetted peatland and the natural mires Degerö and Hälsingfors during the three study years from 2021 to 2023. The bold lines denote the 15‐day running means. The green areas denote the growing season periods.

Over the first three growing seasons following rewetting, NEE at the rewetted peatland demonstrated a clear trend toward increasing net CO_2_ uptake (Figure 3a). Specifically, in the first growing season, there were 35 days with daily net CO_2_ uptake. These uptake periods, primarily between June and August, increased to 80 days in the second year and further to 89 days in the third year. The 90th percentile of maximum daily net CO_2_ uptake increased from −0.28 g C m^−2^ day^−1^ in the first growing season to −0.75 and −0.62 g C m^−2^ day^−1^ in the second and third growing seasons, respectively. Partitioning of NEE into *R*_eco_ and GPP showed that the increase in daily net CO_2_ uptake corresponds to a 7.0% increase in the 90th percentile of the highest daily GPP recorded from 2021 to 2023. The annual NEE at the rewetted peatland was +103 ± 11, +46 ± 6, and +40 ± 7 g C m^−2^ year^−1^ in the years 2021, 2022, and 2023, respectively, with a 3‐year mean of +63 ± 5 g C m^−2^ year^−1^ (Table 2). Comparatively, the Degerö and Hälsingfors mires were sinks of CO_2_ during 112–115 and 86–94 days, respectively, per growing season. The 3‐year mean annual NEE was −59 ± 2 g C m^−2^ year^−1^ and −6 ± 2 g C m^−2^ year^−1^ at the Degerö and Hälsingfors mires, respectively.

**TABLE 2:** Annual net ecosystem carbon balance (NECB) and its component fluxes including net ecosystem CO_2_ exchange (NEE), i.e., the balance of ecosystem respiration (*R*_eco_) and gross primary productivity (GPP), net ecosystem methane (CH_4_) exchange and the lateral export of dissolved carbon (C) at the rewetted peatland and the two natural mires (Degerö and Hälsingfors) during the years 2021–2023.

	Year	Rewetted peatland	Degerö mire	Hälsingfors mire
NEE	2021	103 ± 11	−62 ± 2	−5 ± 2
	2022	46 ± 6	−78 ± 2	−32 ± 2
	2023	40 ± 7	−38 ± 2	17 ± 3
*R*_eco_	2021	395 ± 12	281 ± 8	272 ± 7
	2022	400 ± 7	288 ± 5	263 ± 6
	2023	416 ± 9	289 ± 7	290 ± 11
GPP	2021	−292 ± 10	−343 ± 7	−277 ± 6
	2022	−354 ± 8	−366 ± 4	−295 ± 5
	2023	−376 ± 9	−327 ± 8	−273 ± 10
CH_4_	2021	3.1 ± 0.1	11.1 ± 0.1	8.4 ± 0.1
	2022	5.2 ± 0.2	13.9 ± 0.1	10.7 ± 0.1
	2023	5.8 ± 0.1	13.3 ± 0.1	10.2 ± 0.1
Dissolved C export	2021	9.5 ± 0.1	17.5 ± 0.2	6.1 ± 0.1
	2022	7.8 ± 0.1	9.3 ± 0.1	7.8 ± 0.1
	2023	8.8 ± 0.1	9.5 ± 0.1	8.3 ± 0.1
NECB	2021	116 ± 11	−33 ± 2	10 ± 2
	2022	59 ± 6	−55 ± 2	−14 ± 2
	2023	55 ± 7	−15 ± 2	36 ± 3
GHG balance	2021	489 ± 40	172 ± 8	284 ± 8
	2022	356 ± 23	214 ± 8	268 ± 8
	2023	355 ± 29	339 ± 8	430 ± 12

*Note:* Annual estimates are presented with ± one standard deviation based on a Monte Carlo uncertainty analysis. Except for the greenhouse gas (GHG) balance (in g CO_2_‐eq m^−2^ year^−1^) using global warming potentials of 27 for CH_4_ over a 100 year timeframe (Lee et al. 2023), all fluxes are in the units of g C m^−2^ year^−^1. Note that negative and positive fluxes represent C input (i.e., uptake) into and C output (i.e., emission or lateral export) from the ecosystem, respectively.

During May and June in 2023, which featured below‐normal precipitation, the rewetted peatland remained a significant net CO_2_ sink similar to the net CO_2_ sink periods observed during the same months in 2022 (Figure 3a). In contrast, the net ecosystem CO_2_ uptake was reduced by 30% and 48% at the Degerö and Hälsingfors mires, respectively, relative to the normal years 2021 and 2022. The latter resulted from a 17% and 19% decrease in peak (90th percentile) daily GPP at the Degerö and Hälsingfors mires, respectively, whereas peak daily *R*_eco_ remained similar in all years. Given the reduction in the mire net CO_2_ sink in 2023, the total growing season NEE at the rewetted peatland was not significantly different from that at the Hälsingfors mire (*p* = 0.17) but was still significantly higher than at the Degerö mire (*p* < 0.05).

Our year‐round EC measurements over 3 years further suggest that net CO_2_ loss during the non‐growing season was consistently higher at the rewetted peatland compared with the two mires. Specifically, the average daily NEE during the non‐growing season was +0.24 g C m^−2^ day^−1^ at the rewetted peatland, which is almost two times higher compared with that of +0.15 g C m^−2^ day^−1^ at the Degerö and +0.14 g C m^−2^ day^−1^ at the Hälsingfors mire, respectively.

CH_4_ fluxes exhibited a clear unimodal pattern over the growing seasons at all three sites. The 90th percentile of growing season daily CH_4_ fluxes at the rewetted peatland increased from +35 mg C m^−2^ day^−1^ in the first year to +44 and +55 mg C m^−2^ day^−1^ in the second and third year, respectively (Figure 3d). In comparison, the mean 90th percentile of CH_4_ flux for 2021 and 2022 was 2.5 and 1.9‐fold greater at the Degerö and Hälsingfors mires, respectively, relative to the rewetted peatland. In May and June 2023, which were characterized by lower precipitation, peak CH_4_ emissions in both natural mires decreased significantly (> 20%) compared with the previous 2 years. However, the cumulative growing season emissions were similar (< 1% difference) compared with those of the previous 2 years. Over the 3 years, the mean non‐growing season CH_4_ emission at the rewetted peatland was nearly a magnitude smaller (+1.4 mg C m^−2^ day^−1^) relative to those observed at the Degerö (+10.1 mg C m^−2^ day^−1^) and Hälsingfors (+8.4 mg C m^−2^ day^−1^) mires. Annual CH_4_ emission at the rewetted peatland was +4.7 ± 0.1 g C m^−2^ year^−1^ compared with +12.8 ± 0.1 and +9.8 ± 0.1 g C m^−2^ year^−1^ at the Degerö and Hälsingfors mires, respectively (Table 2).

**FIGURE 4:**
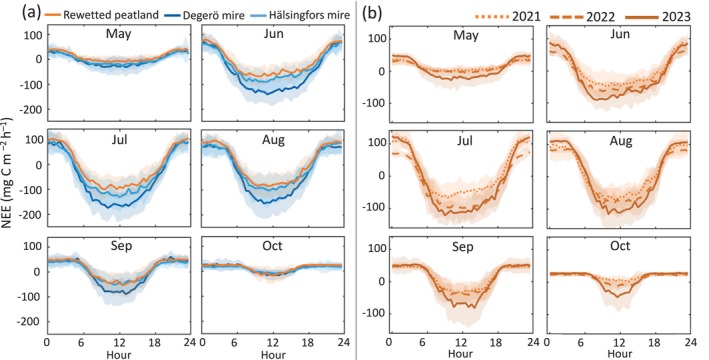
Monthly diel ensembles of the half‐hourly net ecosystem CO_2_ exchange (NEE) of May to October (a) averaged over 3 years (2021–2023) for the rewetted peatland and the two natural mires (Degerö and Hälsingfors) and (b) separated into the three measurement years for the rewetted peatland. The colored lines denote the means, while the shaded area denotes one standard deviation.

At the rewetted peatland, monthly diel ensembles of half‐hourly NEE averaged over the 3 years indicate a mean net CO_2_ uptake of −95 mg C m^−2^ h^−1^ at noon during July (Figure 4a). This was significantly lower than the noon net CO_2_ uptake observed at the Degerö and Hälsingfors mires (−165 and −123 mg C m^−2^ h^−1^, respectively). The nighttime (i.e., PAR < 10 μmol m^−2^ s^−1^) NEE, representing *R*_eco_, peaked at +91 mg C m^−2^ h^−1^ in August at the rewetted peatland (Figure 4a). This was comparable to the mean nighttime peak NEE of 81 and 88 mg C m^−2^ h^−1^ at the Degerö and Hälsingfors mires during the same period. Over the three measurement years at the rewetted site, the mean nighttime peak NEE increased by 17% from the first to the third growing season, whereas a more than two‐fold increase in the daytime peak net CO_2_ uptake was observed across the three growing seasons (Figure 4b).

### 3.3 Responses of Ecosystem‐Scale CO_2_ and CH_4_ Fluxes to Key Environmental Drivers in Rewetted and Natural Peatlands

**FIGURE 5:**
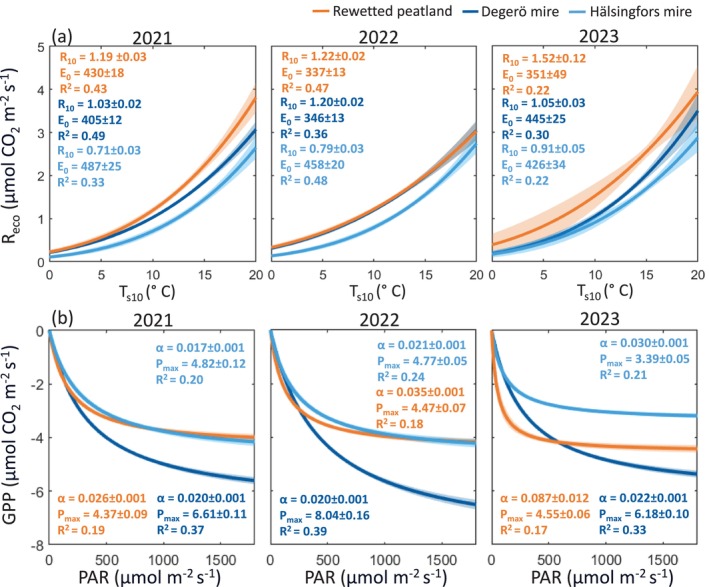
(a) Response of half‐hourly ecosystem respiration (*R*_eco_) to soil temperature at 10 cm depth (*T*_s10_) and (b) response of gross primary productivity (GPP) to photosynthetically active radiation (PAR) during the growing seasons for the rewetted peatland and the two natural mires (Degerö and Hälsingfors) under the three study years. For (a), Lloyd and Taylor equation (1994), where *R*_10_ and *E*_o_ are interpreted as the respiration (μmol CO_2_ m^−2^ s^−1^) at *T*_s_ = 10°C and the activation energy parameter (°C^−1^), respectively, was used to describe the temperature response. For (b), a hyperbolic curve of GPP = −α · PAR · *P*_max_/(α · PAR + *P*_max_), where α and *P*_max_ are interpreted as the initial quantum yield (μmol CO_2_ μmol‐photon^−1^) and maximum ecosystem photosynthesis rate (μmol CO_2_ m^−2^ s^−1^), respectively, was used to describe the light response. The solid line and shaded area denote the mean and the 95% confidence interval of the flux estimates.

The exponential regression between *T*_s10_ and measured *R*_eco_ during nighttime showed that the parameter of basal respiration at 10°C (i.e., *R*_10_) was significantly higher at the rewetted peatland (1.19–1.52 μmol CO_2_ m^−2^ s^−1^) than at the mires (0.71–1.20 μmol CO_2_ m^−2^ s^−1^) over the 3 years (Figure 5a). However, there was no significant difference in the temperature sensitivity parameter (E_0_) between the rewetted and natural peatlands. In the relationship between PAR and GPP, initial quantum yield (α) at the rewetted peatland significantly increased by more than three times from 0.026 μmol μmol^−1^ in 2021 to 0.087 μmol μmol^−1^ in 2023 (Figure 5b). In comparison, α was overall lower, ranging from 0.020 to 0.030 μmol μmol^−1^ and without significant inter‐annual variations at the mires. Averaged over the 3 years, the photosynthetic rate at light saturation (*P*_max_) was higher at the Degerö mire (6.2–8.0 μmol CO_2_ m^−2^ s^−1^) compared with the rewetted peatland (4.4–4.5 μmol CO_2_ m^−2^ s^−1^) and the Hälsingfors mire (3.4–4.8 μmol CO_2_ m^−2^ s^−1^). Furthermore, while *P*_max_ at the rewetted peatland was similar among years, significant inter‐annual variations were observed at the two mire sites, including a reduced *P*_max_ in 2023.

**FIGURE 6:**
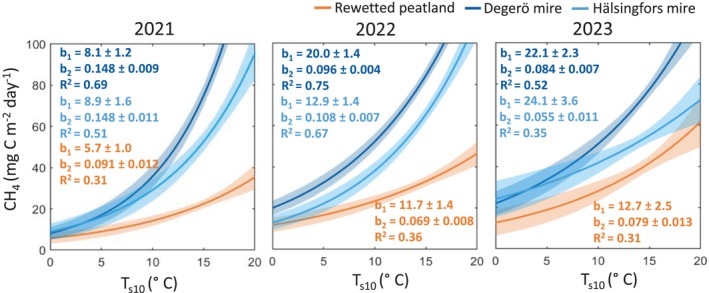
Response of daily sums of methane (CH_4_) emission to daily means of soil temperature at 10 cm depth (*T*_s10_) at the rewetted peatland and the two natural mires (Degerö and Hälsingfors) for the growing seasons 2021 to 2023. An exponential fit with CH_4_ = *b*_1_ · exp. (*b*_2_ · *T*_s10_) was used to describe the *T*_s10_ response. The solid line and shaded area denote the mean and the 95% functional prediction interval of the flux estimates, respectively.

Averaged over the three growing seasons, daily averages of *T*_s10_ and CH_4_ fluxes were significantly correlated (*R* ^2^ > 0.30; *p* < 0.05) at both the rewetted and natural peatlands (Figure 6). The CH_4_ emission at a given temperature increased consistently from 2021 to 2023 at the rewetted peatland, particularly for higher *T*_s_ ranges. The two parameters (*b*_1_ and *b*_2_) of the exponential function were significantly lower at the rewetted peatland than at the natural mires for all study years, except for 2023 when the temperature sensitivity (*b*_2_) remained similar to previous years at the rewetted peatland, while it decreased at the mires. We further noted a negative relationship between CH_4_ emissions and WTL across all the sites, which was likely a confounding result from lower WTL often occurring when *T*_s_ was high (Figure S2).

### 3.4 Dissolved C Export

**FIGURE 7:**
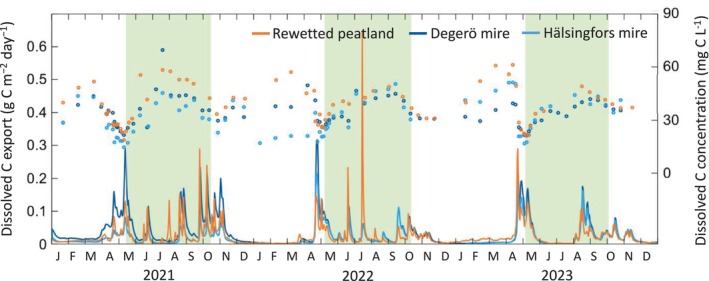
Daily sums of dissolved (organic + inorganic) carbon (C) export at the rewetted peatland and the two natural mires (Degerö and Hälsingfors) from 2021 to 2023. Lines denote dissolved C export (left y‐axis), whereas circular dots denote concentration of dissolved C from manually collected samples (right y‐axis). The green areas denote the growing season periods.

The seasonal variation of the dissolved C (DOC + DIC) export was controlled by the discharge rate at both the rewetted and the natural peatlands (Figure 7). During the first growing season, the dissolved C concentration at the rewetted peatland was significantly higher (*p* < 0.05) than at the two natural mires, but similar in the subsequent two growing seasons. Averaged over the 3 years, the annual discharge at the rewetted peatland was 237 mm year^−1^, being lower than at the Degerö (375 mm year^−1^) and Hälsingfors mires (256 mm year^−1^). The annual export of dissolved C from the rewetted peatland was 8.7 ± 0.1 g C m^−2^ year,^−1^ which was significantly lower than at the Degerö mire (12.1 ± 0.2 g C m^−2^ year^−1^) and higher than that at the Hälsingfors mire (7.4 ± 0.2 g C m^−2^ year^−1^) but similar to their average (9.8 g C m^−2^ year^−1^) (Table 2).

### 3.5 Net Ecosystem Carbon Balance and Total GHG Emissions

After integrating the terrestrial and dissolved C flux components, we estimated the rewetted peatland to be a net C source with a NECB of +116 ± 11, +59 ± 6, and +55 ± 7 g C m^−2^ year^−1^ in the years 2021, 2022, and 2023, respectively, suggesting that the C source strength decreased by about half from 2021 to 2023 (Table 2). When comparing absolute fluxes for evaluating the relative importance of the NECB components, NEE accounted on average for 83%, while CH_4_ emissions and dissolved C export contributed about 6% and 11%, respectively, to the total absolute C flux. When considering the 27 times higher warming potential of CH_4_ over a 100‐year time frame (Lee et al. 2023), the total GHG emission was 400 ± 18 g CO_2_‐eq m^−2^ year^−1^ averaged over the 3 years at the rewetted peatland.

In comparison, the Degerö mire consistently acted as an annual C sink with a 3‐year mean NECB of −34 ± 2 g C m^−2^ year^−1^, while the Hälsingfors mire was a sink during 2021 and 2022, but a source during 2023, resulting in a 3‐year mean NECB of +11 ± 2 g C m^−2^ year^−1^ (Table 2). NEE contributed 70% and 29% to the total C flux (i.e., the sum of absolute NECB component fluxes) at the Degerö and Hälsingfors mires, respectively. Thus, the relative importance of CH_4_ (15% and 41%) and dissolved C export (14% and 31%) averaged for the two mires was greater compared With the rewetted peatland. Over a 100‐year timeframe, the mires were also GHG sources to the atmosphere, with annual emissions of 242 ± 5 and 327 ± 8 g CO_2_‐eq m^−2^ year^−1^ at the Degerö and Hälsingfors mires, respectively.

### 3.6 Divergence in C and GHG Balance Components of Rewetted and Natural Peatlands

**FIGURE 8:**
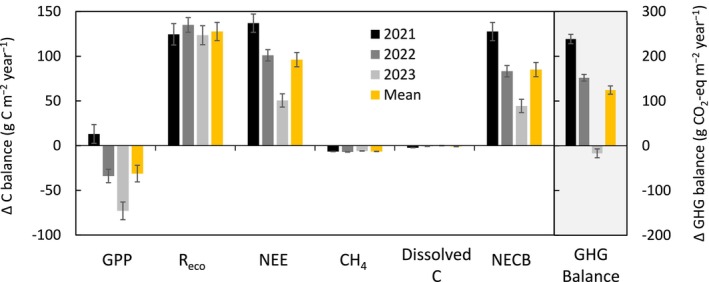
Difference in annual net ecosystem carbon balance (NECB) including its component fluxes, as well as the greenhouse gas (GHG) balance based on 100‐year global warming potentials at the Trollberget rewetted peatland compared with the average values from Degerö and Hälsingfors natural mires over 2021 to 2023. Positive values indicate a negative climate impact due to either increased carbon emissions or decreased carbon uptake at the rewetted peatland compared with the mires. C balance values are referenced on the left y‐axis (in g C m^−2^ year^−1^), while the GHG balance is presented on the right y‐axis (in g CO_2_‐eq m^−2^ year^−1^). Error bars denote one standard deviation based on a Monte Carlo uncertainty analysis. See Table 2 for a description of flux abbreviations.

The annual GPP at the rewetted peatland was statistically similar to the average of the two mires during the first year following rewetting, but greater (i.e., more negative) by 24 and 76 g C m^−2^ year^−1^ (equal to 7% and 25%) in the second and third year (Figure 8). In comparison, annual *R*_eco_ was consistently higher by 119–127 g C m^−2^ year^−1^ (equal to 43%–45%) compared with the average of the mires. The differences in annual NEE decreased from 137 to 51 g C m^−2^ year^−1^ (3‐year mean: 94 g C m^−2^ year^−1^) from 2021 to 2023, with a direction from net CO_2_ uptake at the mires toward net CO_2_ loss at the rewetted peatland. Annual CH_4_ emissions were 51%–68% lower at the rewetted peatland compared with the mires. In comparison, the differences in annual dissolved C export were 10%. Overall, the difference in NECB of the rewetted and natural peatlands was on average 88 g C m^−2^ year^−1^ across the three study years. This divergence in NECB was primarily driven by the differences in NEE. The annual GHG emissions (i.e., in CO_2_‐eq, over a 100‐year time frame) at the rewetted peatland were on average 51% higher than at the mires. However, in 2023, the GHG balance between the rewetted peatland and the mires was statistically similar, mainly due to a reduced difference in NEE.

## 4 Discussion

### 4.1 A Recently Rewetted Boreal Minerogenic Peatland Acts as a Net C Source

In this study, we integrated terrestrial and aquatic C fluxes into annual ecosystem‐scale C balances for a recently rewetted peatland and two nearby natural mires in northern Sweden over 3 years. Our main finding suggests that this rewetted peatland was a C source (+55 to +116 g C m^−2^ year^−1^) during the initial 3 years following rewetting, whereas the two undisturbed mires functioned as a C sink (−34 ± 2 g C m^−2^ year^−1^ at the Degerö mire) or remained close to C‐neutral (+11 ± 2 g C m^−2^ year^−1^ at the Hälsingfors mire). We also observed considerable differences in the relative importance of the various NECB components. Thus, our findings challenge the simplifying assumption of similar C cycles in natural and rewetted peatlands, which is inherent in the current default EFs for rewetted peatlands in the boreal region. Specifically, our results indicate a bias in the short‐term climate benefit of rewetting measures as suggested by the application of EFs, given that the EFs for rewetted peatlands are primarily based on data from mires and therefore imply an immediate C sink function following rewetting.

At present, empirical estimates of full annual C balances are lacking for rewetted minerogenic peatland systems in the boreal region. However, similar to our findings, a rewetted fen (previously drained for grassland use) in north‐eastern Germany was also reported to act as an annual C source for more than one decade after rewetting (Kalhori et al. 2024). Our results also align with studies in rewetted bogs after peat extraction, reporting net C losses during the first decade after rewetting (Petrone et al. 2001; Järveoja et al. 2016; Strack and Zuback 2013). In comparison, Renou‐Wilson et al. (2019) reported that a rewetted nutrient‐poor cutover bog in Ireland was a net C sink (−29 g C m^−2^ year^−1^), while a rewetted nutrient‐rich industrial cutaway site had a near‐neutral C balance (+6 g C m^−2^ year^−1^) in the first year. Overall, our findings combined with results from previous studies highlight that recently rewetted peatlands commonly act as C sources immediately after rewetting, with the source magnitude depending strongly on local site conditions related to soil fertility, hydrology, and prior land use.

It is further noteworthy that since our EC measurements did not cover the period before rewetting, our study cannot provide insights into the potential of rewetting for improving the C balance relative to the drained conditions. For instance, Komulainen et al. (1999) found that rewetting increased the growing season net C uptake relative to drained forested conditions, with differences noted between nutrient‐rich and nutrient‐poor sites. Furthermore, studies reporting annual C balances for drained peatland forests in the nutrient‐poor boreal region suggest that at the ecosystem scale, these might act as considerable C sinks ranging from −105 to −570 g C m^−2^ year^−1^ (Lohila et al. 2011; Minkkinen et al. 2018; Tong et al. 2024; Ojanen et al. 2013). Thus, combined with our results, this indicates that rewetting of such areas has initially a negative climate impact. However, while beyond the focus of our study, the central questions to further explore are whether rewetting can effectively mitigate the C loss from the peat layer and within which timeframe a rewetted peatland may resume a C sink and net climate cooling function (Ojanen and Minkkinen 2020; Laine et al. 2024).

Our findings further revealed strong temporal dynamics in the NECB and its components during the initial years following the rewetting of a boreal forested peatland. Specifically, the decrease in net annual CO_2_ emissions by 56% and the concurrent increase in CH_4_ emissions by 87% during the first 3 years suggest a period of considerable transformation in the biogeochemical processes in recently rewetted minerogenic boreal peatlands. Recent studies from the temperate region suggest that the initial phase of transformation may take several decades for a rewetted system before it may resemble close‐to‐natural states (Kreyling et al. 2021) and more than 13 years for GHG emissions to reach levels comparable to the IPCC default EFs for rewetted peatlands (Kalhori et al. 2024). A dynamic recovery phase, including initial net C losses, has also been reported for rewetted bogs during the first decade following peat extraction in Canada (Petrone et al. 2001; Strack and Zuback 2013) and during at least one decade following the rewetting of an afforested drained peatland in Scotland (Hambley et al. 2019). Although our study was limited to the initial 3 years after rewetting, long‐term monitoring will be needed to evaluate and compare the recovery trajectory of this rewetted minerogenic peatland relative to these contrasting systems reported in the literature. Altogether, however, our findings, combined with those from other studies, call for dynamic EFs to better represent the C sink‐source development and associated short‐term climate impact for recently rewetted peatlands.

### 4.2 NECB Component Fluxes Differ in Rewetted and Natural Boreal Peatlands

The reduction of net CO_2_ emissions during the first 3 years following rewetting was mainly due to the increase in GPP rather than a decrease in *R*_eco_. The overall increase and inter‐annual patterns in GPP corresponded closely to those of the growing season NDVI, suggesting an immediate response of the vegetation communities to rewetting. Although quantitative estimates of changes in vegetation biomass were not available in our study, an increase in the abundance of cottongrass establishing across the site (and predominantly along the in‐filled ditches) was noted visually during the three study years. Similarly, Purre et al. (2019) suggested that the establishment of vascular plants with higher photosynthetic capacity relative to mosses contributed to increased GPP in rewetted peatlands. It is further noteworthy that the GPP at our rewetted site was little affected by the 2023 summer drought, compared with the mires where the GPP was considerably reduced during this period. This is particularly surprising given that we observed lower WTL minima in the rewetted peatland as a result of altered peat hydraulic properties following over a century of drainage. This might be due to the greater presence of remnant dry‐adapted shrubs and sedges with long‐reaching roots that can access deeper water during drought. In comparison, the mires are dominated by moss communities which depend on capillary forces, which decrease their efficiency in accessing water during drought periods. The greater drought resistance of our rewetted minerogenic peatland contrasts with the enhanced drought sensitivity of GPP reported from rewetted peat extraction areas where reduced peat pore connectivity limits water access for the recently restored moss communities (McCarter and Price 2014; He et al. 2024). Altogether, this underscores the complex interplay between peatland drainage history, hydrology, vegetation recovery, and C dynamics following the rewetting of drained peatlands.

In contrast to the increase in GPP, the annual *R*_eco_ remained similar at the rewetted peatland over the three measurement years. This implies that an increase in autotrophic respiration in response to increasing GPP (possibly combined with heterotrophic respiration from decaying harvest residues) might have partly counterbalanced the likely reduction in soil heterotrophic respiration in these initial years. A similar change in the ratio of heterotrophic to autotrophic respiration was previously observed in a temperate rewetted peatland (Nyberg et al. 2022). The 42%–50% higher *R*_eco_ at the rewetted peatland relative to that at the mires appeared surprising given that both systems had similar WTL. However, the fresh C input from decomposing plant material after machinery disturbance and residuals (i.e., slash and decaying roots) from tree harvest likely stimulated the microbial decomposition of soil organic C (Marinier et al. 2004; Mäkiranta et al. 2012), thereby contributing to an enhanced *R*_eco_. Altogether, this underscores that the overall rewetting effect on *R*_eco_ requires a detailed understanding as it includes contributions from several simultaneous processes that are likely to shift over time.

The higher annual *R*_eco_ at the rewetted peatland was also supported by a substantial contribution from higher *R*_eco_ during the non‐growing season compared with the natural mires. This is possibly due to the large amount of decaying slash and root residues following the rewetting combined with tree harvest, which may act as an important source for winter CO_2_ emissions (e.g., Mäkiranta et al. 2012; Korkiakoski et al. 2019). Insights into the non‐growing season C fluxes are particularly crucial in the boreal region, where the winter period may last for approximately 6 months. Thus, while previous chamber‐based studies have provided valuable information on the growing season C balance, our study promotes the need for year‐round EC measurements to gain new insights into the whole‐year dynamics of C fluxes and the annual C sink‐source function of rewetted boreal peatlands.

The nearly doubling of CH_4_ emission within the first 3 years following rewetting demonstrates its considerable potential for counterbalancing the desired mitigation effect from reduced CO_2_ emissions. The CH_4_ emissions from our studied rewetting site were about twice as high compared with those (1.6–3.5 g C m^−2^ year^−1^) reported in the first 2 years after rewetting and re‐introduction of *Sphagnum* spp. mosses in a cut‐away peatland in boreal Finland (Tuittila et al. 2000a). This suggests that, compared with peat extraction sites, the rewetting of peatlands drained for forestry may cause higher CH_4_ emissions as the presence of vegetation already before rewetting may provide substrate and favorable environments to facilitate methanogen establishment (Urbanová and Bárta 2020). Furthermore, the rapid establishment of cottongrass noted across the site after rewetting may provide an additional transport channel for CH_4_ emission through their aerenchymatic plant tissue (Marinier et al. 2004). Indeed, large CH_4_ emissions (~40 g C m^−2^ day^−1^ during the growing season) have been reported in southern boreal Finland in 11‐ to 17‐year‐old rewetted sites, being 35 times higher than in a nearby natural mire (Koskinen et al. 2016). This highlights the potential for growing season CH_4_ emissions to further increase in rewetted peatlands even beyond the levels observed in natural mires. On the other hand, our year‐round EC measurements also revealed that the winter CH_4_ emissions were by almost one magnitude smaller in the rewetted peatland compared with the mires. This highlights the importance of conducting year‐round CH_4_ measurements (i.e., by the use of EC) to capture the full annual C and GHG budgets, particularly in the boreal region where fluxes during the long (i.e., ~6 months) winter period may considerably affect the annual balance. Overall, given its stronger GWP, the contribution of CH_4_ to the overall GHG balance in our study was comparable to that of CO_2_ over a 100‐year timeframe. This emphasizes the significant role of CH_4_ in regulating the climate impact of rewetted peatlands and calls for an improved understanding of CH_4_ emission dynamics following rewetting.

The higher annual dissolved C export at the rewetted peatland during the first year, relative to the mires, was likely caused by the immediate increase in microbial decomposition associated with the new input of organic materials due to disturbance and tree harvest occurring during the rewetting activities. Furthermore, a rise in WTL following rewetting may create hotspots of high DOC concentrations due to high rates of anaerobic decomposition (Fenner and Freeman 2011). Increased DOC levels immediately after rewetting have also been reported for oligotrophic peatlands in boreal Finland, primarily due to the flushing of highly decomposed surface peat layers that remained oxic for decades as a result of drainage (Menberu et al. 2017). We caution that some of the dissolved C originated via minerogenic inflow from the upland forest area located within the catchment. However, a previous study of the site hydrology suggests that such effects on the C export are likely small (Karimi et al. 2024). Overall, given that the aquatic C export differed between the rewetted and natural peatlands only during the first year, we conclude that the lateral C flux plays a minor role in the divergence of the NECB between recently rewetted and natural peatlands.

### 4.3 Different Environmental Response Functions in Rewetted and Natural Peatlands

Our results further indicate that the vertical fluxes of CO_2_ and CH_4_ responded differently to key environmental controls at the rewetted peatland compared with the natural mires. The higher *R*_10_ value in the *T*_s_‐*R*_eco_ response function at the rewetted peatland suggests elevated *R*_eco_ across all *T*_s_ ranges during the initial years post‐rewetting. This can be attributed to increased inputs of fresh organic matter in rewetted peatlands, which provide easily decomposable material that enhances microbial activity under all temperatures (Straková et al. 2011). In contrast, natural mires contain older organic matter that is more resistant to decomposition (Urbanová et al. 2018). However, it is important to note the difference in the *T*_s_‐*R*_eco_ response sensitivity parameter (*E*_o_) between the two mires, with the one at the Hälsingfors mire resembling more closely *E*_o_ at the rewetted peatland. This similarity in *E*_o_ may be explained by the comparable C:N ratio between the rewetted peatland (45.5) and the Hälsingfors mire (45.0), compared with the Degerö mire (66.5). The C:N ratio as a measure of peat quality (i.e., peat constituents and their nutrient contents) has been shown to explain variations in the temperature sensitivity of the decomposing communities in response to substrate quality (Briones et al. 2014; Liu et al. 2024). Therefore, while the recently rewetted peatland exhibits higher baseline respiration due to increased litter input, the temperature sensitivity of respiration might more strongly depend on soil fertility.

The observed divergence in the initial photosynthetic light‐use efficiency (i.e., the alpha parameter) demonstrates a functional difference between rewetted and natural peatlands, with consequences for their ability to assimilate atmospheric CO_2_. Differences in plant functional composition (i.e., the fraction of moss and vascular plants) likely explain this divergence in their light‐use efficiency (Peichl et al. 2018). Furthermore, the reduction of *P*_max_ at the mire sites in 2023 helps to explain the lower GPP during the dry summer, relative to the rewetted site where *P*_max_ remained unaffected. Our study thus corroborates previous findings that highlight distinct drought‐coping mechanisms in rewetted peatlands compared with natural mires (e.g., Beyer et al. 2021). However, the long‐term shift toward wet‐adapted species following rewetting could potentially diminish this resilience over time.

We further observed a lower temperature response of CH_4_ fluxes at the rewetted peatland, relative to the mires. This difference is likely due to less developed methanogenic populations at the recently rewetted peatland (Urbanová and Bárta 2020). During the dry growing season of 2023, the parameter representing sensitivity to temperature change (*b*_2_ in the exponential equation) remained consistent at the rewetted peatland compared with the average of the previous 2 years. However, at the natural mires, this parameter decreased significantly, indicating that CH_4_ emissions at the rewetted peatland are more resilient to drought conditions than those at the natural mires. This could be associated with reduced development of cottongrass at the natural mires during drought, consequently reducing CH_4_ transport via their aerenchymatic plant tissue into the atmosphere (Greenup et al. 2000). Thus, the lower temperature sensitivity of CH_4_ emissions combined with greater resilience to drought further highlights a functional difference in the biogeochemistry of recently rewetted peatlands compared with natural mires.

Overall, these findings demonstrate that rewetting may lead to distinct environmental responses in the C cycle compared with natural mires. These differences highlight the need for developing ecosystem models that accurately represent the new response functions of rewetted peatlands. Thus, we consider continued field‐based monitoring combined with improved model simulations as the key prerequisites for accurately evaluating the effectiveness of peatland rewetting strategies for mitigating climate change.

### 4.4 On the Path Toward Convergence?

Altogether, our findings reveal numerous differences in the C balance and ecosystem functioning of our rewetted and natural peatland sites. Namely, the non‐growing season and annual balances of NEE and its component fluxes, as well as non‐growing season and annual CH_4_ emissions, the annual NECB and 3‐year mean GHG balance differed considerably. Furthermore, functional differences including contrasting WTL dynamics and different responses of *R*_eco_, GPP, and CH_4_ to key environmental drivers were noted. However, we also observed some similarity between the rewetted and natural systems. This includes a clear trend in annual NEE at the rewetted site toward mire NEE levels, with similar growing season NEE between the rewetted site and the Hälsingfors mire observed in 2023. In addition, we noted similar C export after the first year as well as a similar GHG balance in 2023. However, we show that the similarities in growing season NEE and annual GHG balance in 2023 were primarily due to changes in the mire functioning during the dry summer, rather than the NEE at the rewetted site reaching the levels of the mire NEE during the normal years (2021 and 2022). We further show that the choice of mire reference may affect the comparison with the rewetted peatland. This study was able to include two mire stations, which are representative of fen‐type mires in boreal Sweden (Nilsson et al. 2001), yet featured differences within the range of boreal mires. Since extensive replication is commonly not possible in ecosystem‐scale studies based on EC measurements, this highlights the need to consider such variations even within a given peatland type and its implication for the baseline in comparison studies.

Given the limited period of the initial 3 years covered in this study, it remains elusive if these observed similarities are transient or the first signs of a convergence of the C cycles at the rewetted and natural peatlands. On the one hand, knowledge from rewetted peat extraction and afforested peatland sites (Nugent et al. 2018; Hambley et al. 2019; Renou‐Wilson et al. 2019) suggests that the recovery of the C sink function may take 10–15 years. Possibly, the recovery period may be even shorter relative to that of a rewetted peat extraction site, given that ground vegetation is already present when rewetting a long‐term drained peatland forest. On the other hand, there is evidence that it may take several decades for other ecosystem functions of a rewetted peatland to resemble those of undisturbed mires (Kreyling et al. 2021). Thus, continued monitoring will be critical to advance our understanding of the trajectory and recovery time of the C sink function following the rewetting of boreal peatland forests.

## Author Contributions

**Cheuk Hei Marcus Tong:** conceptualization, data curation, formal analysis, investigation, methodology, visualization, writing – original draft. **Matthias Peichl:** conceptualization, data curation, methodology, writing – review and editing. **Koffi Dodji Noumonvi:** conceptualization, data curation, formal analysis, investigation, methodology, visualization, writing – original draft. **Mats B. Nilsson:** data curation, methodology, resources, writing – review and editing. **Hjalmar Laudon:** data curation, methodology, resources, writing – review and editing. **Järvi Järveoja:** conceptualization, data curation, funding acquisition, investigation, methodology, project administration, resources, supervision, writing – review and editing.

## Conflicts of Interest

The authors declare no conflicts of interest.

## Supporting information


**Figure S1.** Coverage of 30‐min eddy covariance data for (a, b) Trollberget rewetted peatland, (c, d) Degerö, and (e, f) Hälsingfors natural mires. Methane (CH_4_) fluxes during the study period. Panels (a), (c), and (e) represent data for carbon dioxide (CO_2_), while panels (b), (d), and (f) represent data for CH_4_.
**Figure S2.** Correlation of water table level (WTL) with (a–c) methane (CH_4_) emissions and (d–f) temperature‐normalized CH_4_ emissions, i.e., residuals from the soil temperature (*T*_s_)–CH_4_ relationship, for rewetted peatland and natural mire sites during the growing season across three study years. Data samples were grouped into daily means. Dots represent daily mean values, and lines indicate the best‐fit line.
**Figure S3.** Scatter plot of 10‐fold cross‐validation highlighting the performance of the (a) XGBoost gapfilling models for carbon dioxide (CO_2_) and (b) random forest gapfilling models for methane (CH_4_), applied at the rewetted peatland and the two natural mires (Degerö and Hälsingfors). The red and gray lines denote the best‐fitted line of the distribution, and the 1: 1 reference line, respectively.

## Data Availability

The data that support the findings of this study are openly available in Dryad at http://doi.org/10.5061/dryad.9kd51c5v9.
