# Supplementary material for: The Carbon Balance of a Rewetted Minerogenic Peatland Does Not Immediately Resemble That of Natural Mires in Boreal Sweden
Source: Glob Chang Biol. 2025 Apr 8;31(4):e70169. doi: 10.1111/gcb.70169 (PMC11979567; doi:10.1111/gcb.70169)
Supplement: Supplementary file 1 — Figure S1. Coverage of 30‐min eddy covariance data for (a, b) Trollberget rewetted peatland, (c, d) Degerö, and (e, f) Hälsingfors natural mires. Methane (CH4) fluxes during the study period. Panels (a), (c), and (e) represent data for carbon dioxide (CO2), while panels (b), (d), and (f) represent data for CH4. Figure S2. Correlation of water table level (WTL) with (a–c) methane (CH4) emissions and (d–f) temperature‐normalized CH4 emissions, i.e., residuals from the soil temperature (T s)–CH4 relationship, for rewetted peatland and natural mire sites during the growing season across three study years. Data samples were grouped into daily means. Dots represent daily mean values, and lines indicate the best‐fit line. Figure S3. Scatter plot of 10‐fold cross‐validation highlighting the performance of the (a) XGBoost gapfilling models for carbon dioxide (CO2) and (b) random forest gapfilling models for methane (CH4), applied at the rewetted peatland and the two natural mires (Degerö and Hälsingfors). The red and gray lines denote the best‐fitted line of the distribution, and the 1: 1 reference line, respectively. [file GCB-31-e70169-s001.docx]

**Supporting Information (S)**


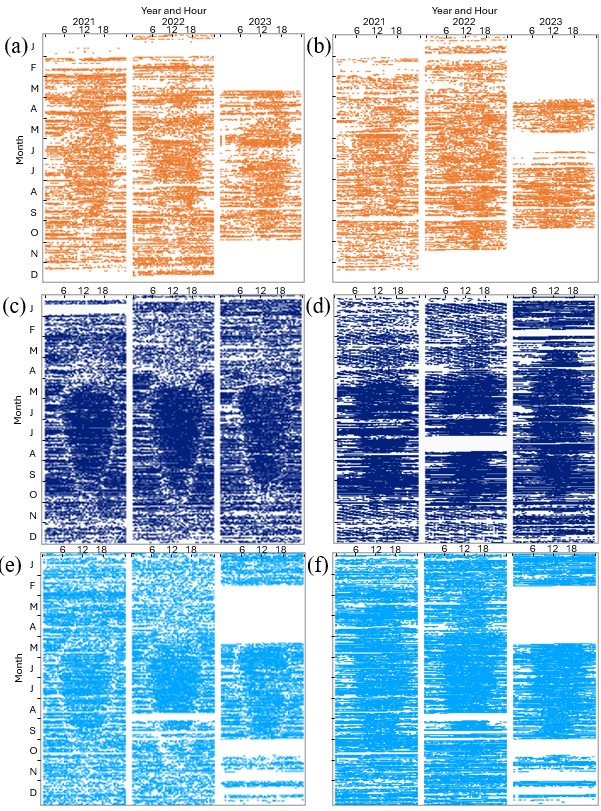


**Figure S1.** Coverage of 30-min eddy covariance data for (a-b) Trollberget rewetted peatland, (c-d) Degerö and (e-f) Hälsingfors natural mires. Methane (CH_4_) fluxes during the study period. Panel (a), (c) and (e) represent data for carbon dioxide (CO_2_), while panel (b), (d) and (f) represent data for CH_4_.


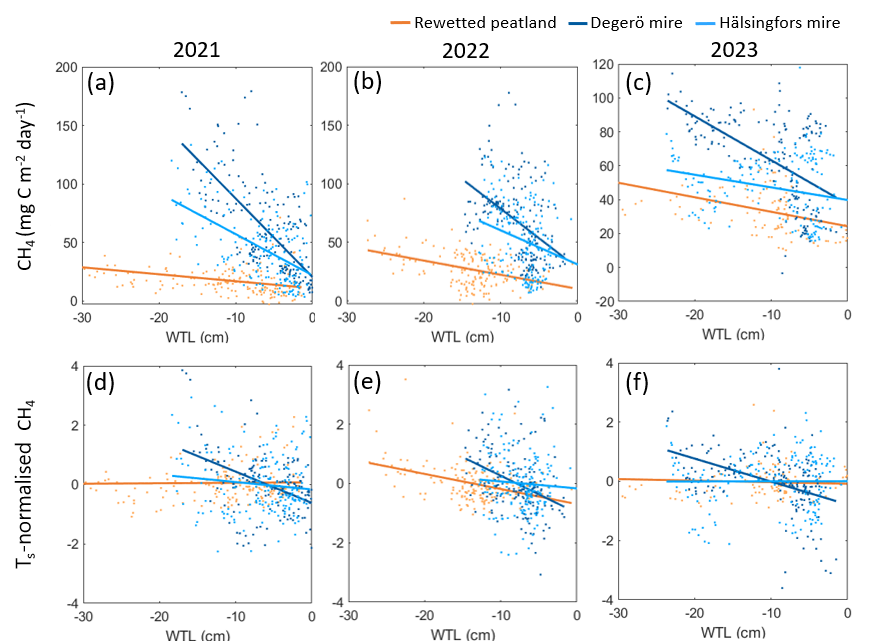


**Figure S2.** Correlation of water table level (WTL) with (a-c) methane (CH_4_) emissions and (d-f) temperature-normalized CH_4_ emissions, i.e., residuals from the soil temperature (T_s_) - CH_4_ relationship, for rewetted peatland and natural mire sites during the growing season across three study years. Data samples were grouped into daily means. Dots represent daily mean values, and lines indicate the best-fit line.


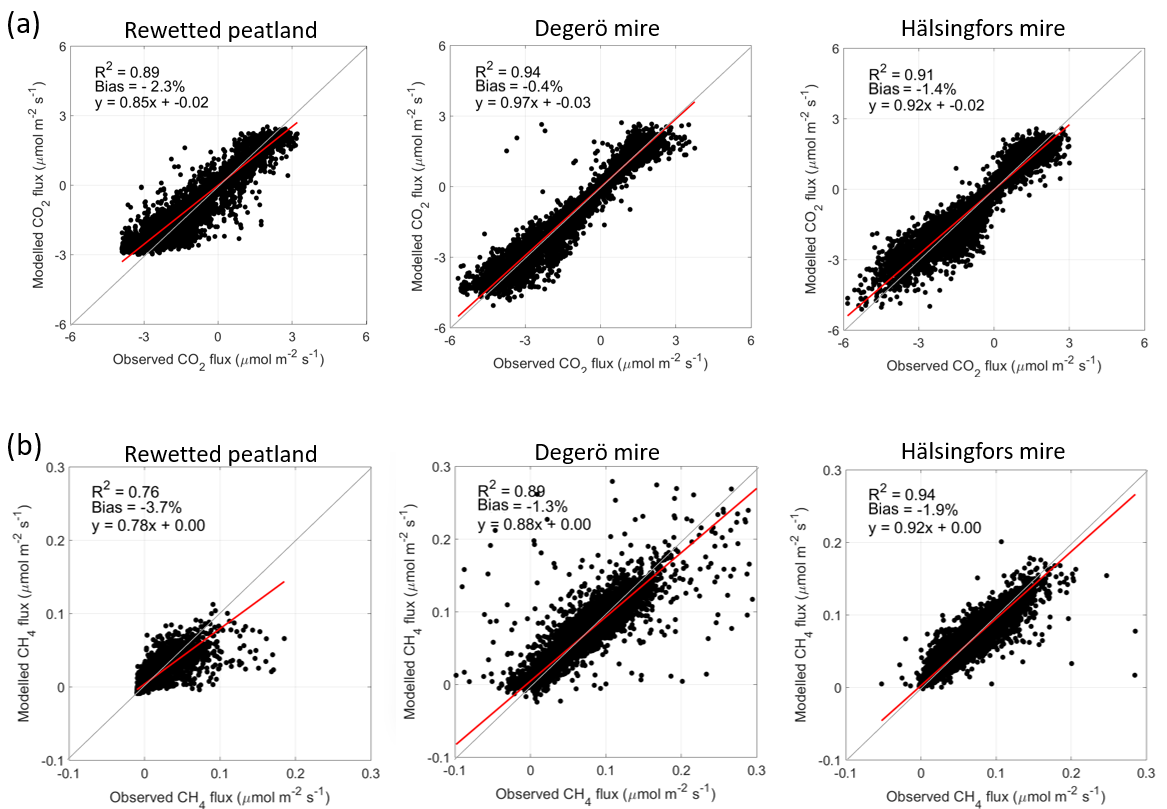
**Figure** **S3.** Scatter plot of 10-fold cross validation highlighting the performance of the (a) XGBoost gapfilling models for carbon dioxide (CO_2_) and (b) Random Forest gapfilling models for methane (CH_4_), applied at the rewetted peatland and the two natural mires (Degerö and Hälsingfors). The red and gray line denote the best-fitted line of the distribution, and the 1: 1 reference line, respectively.
